# A retrospective medical record review of patients’ decisions to utilise or refrain from brief admission

**DOI:** 10.1007/s44192-026-00479-4

**Published:** 2026-06-15

**Authors:** E. Tauson, R. Wärdig, S. Hultsjö

**Affiliations:** 1 Department of Psychiatry, Ryhov County Hospital, 551 85 Jönköping, Sweden; 2 Department of Health, Medicine and Caring Sciences. Division of Nursing and Reproductive Health, Linköping University, Linköping, Sweden

**Keywords:** Brief admission, Convergent mixed method design, Health promotion, Psychiatry, Retrospective medical chart review

## Abstract

**Background:**

Brief Admission (BA) allows patients to independently access short-term psychiatric care during periods of acute stress or perceived threat, offering a safe and structured respite. BA has been associated with reduced self-harm and hospital readmissions. Understanding the factors behind individual differences in BA use and experience is therefore essential.

**Aim:**

To examine patients’ decisions to utilise or refrain from brief admission.

**Methods:**

A retrospective review of 66 medical records (representing 47% of all cases with BA agreements) was conducted using a convergent mixed-methods design. Conventional qualitative content analysis was performed, and the resulting categories were summarized using frequencies and percentages based on the number of records assigned to each category.

**Results:**

According to the medical records, 49% of the patients had never utilised BA, while 23% reported using it less than once per year. The patients perceived BA as beneficial in managing their mental health. By maintaining meaningful activities and self-care routines, the patients were able to preserve behaviours that contributed to their well-being during BA.

Identified challenges that could hinder the utilisation of BA included difficulties in assessing appropriate timing, staff attitudes, fear of detention, concerns about contact with other individuals with mental health problems, and the need for social support to facilitate engagement with BA. Despite BA’s aim of offering a safe environment, it may also trigger past traumatic or negative experiences in patients and relatives, potentially limiting its use.

**Conclusions:**

Access to BA may enhance patient well-being. Given the overlapping challenges patients often face, individually tailored strategies, supported by existing networks and outpatient staff, can help determine when BA or other interventions are appropriate.

## Introduction

More than 720,000 people die by suicide each year globally (World Health Organization, [67]). The prevalence of non-suicidal self-injury (NSSI) is understandably significantly higher. The average lifetime prevalence of NSSI is estimated at approximately 22% in the general population. Repetitive forms of NSSI are more common than single episodes. Clinically, individuals with NSSI often show health-compromising behaviours such as self-cutting, self-burning, aggression, risky sexual activity and eating disorders [34, 46]. Ingesting toxic substances is less common, reported in only 1% of cases [70]. In 2022, nearly 7,000 individuals aged 15 and older were admitted to inpatient psychiatric care in Sweden due to self-harm or suicide attempts. The statistics encompass confirmed cases of self-harm, suicide attempts, and injury events of undetermined intent [48]. Various restrictive interventions are employed to manage impulsive self-harming and suicidal behaviours, including prolonged psychiatric hospitalisation, coercive measures, and involuntary admissions for life-sustaining purposes [17, 43].

Patients who engage in self-harming behaviours are recommended therapeutic interventions such as Dialectical Behaviour Therapy (DBT), Mentalisation-Based Therapy and Schema Therapy. These approaches are supported by substantial empirical evidence regarding their efficacy in alleviating self-harming behaviour [33, 52]. However, they are not implemented in clinical practice to the extent that would be clinically feasible or desirable [33]. Although psychotherapeutic treatment has been employed for many years, it is well established that patients with self-harming behaviours are high consumers of psychiatric inpatient care [45, 46, 71]. Reasons for admission to psychiatric inpatient care include, for example, severe self-harm, intentional self-poisoning or injury with either suicidal or non-suicidal intent [44], as well as anxiety, chaotic life circumstances and suicidality [32]. Within inpatient settings, there has long been a lack of evidence-based approaches for treating these psychiatric disorders, often resulting in prolonged admissions and the use of coercive measures that undermine personal autonomy [44].

To address this issue, Brief Admission (BA) was developed as a crisis intervention strategy for situations in which patients experience intense anxiety, urges to self-harm, or suicidal ideation [11, 19, 26, 36]. BA may be utilised as a complement to outpatient treatment, either as a preventive measure or as a crisis intervention to avert self-harm [1]. Persons with access to BA always have the option of seeking emergency psychiatric care if needed [1, 31]. BA allows patients to voluntarily seek short-term inpatient care, for a duration of three to seven days [18, 19, 49, 57, 59]. The purpose is to provide patients with a reprieve during periods of heightened threat or stress, thereby supporting their ability to manage their mental health in a safe environment [9]. BA is designed to enhance autonomy, participation, and self-awareness, as well as to strengthen patients’ capacity to recognise early signs of psychological deterioration [40]. This, in turn, is expected to reduce the need for and shorten the duration of inpatient admissions [8]. BA has been shown to reduce self-harming behaviours and readmissions over the long term [7, 11, 58]. It differs from standard inpatient care with regard to both structure and content. The possibility for patients to initiate admission autonomously, together with access to individually tailored support, strengthens their capacity to cope with everyday life [13, 49, 64]. There is now substantial empirical support for the application of BA in different psychiatric diagnoses, including Borderline Personality Disorder (BPD) [18], severe mental illness, with or without problematic substance use [4, 49, 59], schizophrenia, bipolar disorder [47], and eating disorders [56].

Currently, BA is offered within both adult psychiatric services ([1, 7, 9, 13, 35, 36, 49]), and child and adolescent psychiatric services [26, 27, 31, 45, 70]. Research indicates that patients who utilise BA, their relatives, and healthcare professionals working with the model report positive experiences. Patients describe increased autonomy and involvement in their care, while staff report reduced work-related stress and a shift in focus from symptom management to patients’ health and recovery potential [8, 13, 37]. Furthermore, symptoms of anxiety and depression decrease, and health-related quality of life improves following the use of BA [10]. BA is also perceived as an effective nursing intervention that fosters self-determination and self-care, thereby facilitating individuals’ ability to maintain everyday routines, employment, and interpersonal relationships more effectively [7, 9, 13, 40, 63]. Outpatient nurses state that the availability of BA reassures them, as they know that patients can obtain care promptly if needed [1, 35]. Psychotherapeutic interventions in outpatient settings are perceived as more effective, and their benefits are more enduring when patients also have access to BA [1]. Family members of patients with access to BA describe the model as a source of hope and a helpful tool for maintaining daily routines, and state that it contributes to improved family relationships [23, 24, 39]. Conversely, when patients are denied access to BA, family members have reported feelings of betrayal and a loss of trust in psychiatric services [23, 24, 39]. BA has been shown to be associated with a reduction in the length of individual hospital stays, though not in the total number of inpatient days [11]. This suggests a potential shift from prolonged inpatient care to increased utilisation of outpatient services. Cost-effectiveness analyses have indicated that BA is associated with an annual cost reduction of between €4,600 and €4,800 per patient [38]. The greatest savings were observed among individuals who had experienced more than 180 inpatient days before gaining access to BA. Furthermore, BA was significantly associated with an increase in quality-adjusted life years for patients with self-harming behaviours and suicidality. Patients report that access to BA has led to increased independence, reduced self-harming behaviours, decreased time spent in inpatient care, and improved quality of life [63, 64]. Given that BA promotes health on multiple levels and enhances the sense of safety and hope among both patients, clinicians and family members, continued research in this area is warranted to expand access to and utilisation of BA. In a recently published study, Lindkvist et al. [38] emphasise the need for future research to explore explanatory factors underlying individual variation in the use and effectiveness of BA, an objective that underpins the present study.

### Aim

To examine Patients’ Decisions to Utilise or Refrain from Brief Admission.

## Methods

A retrospective medical record review was conducted [53] using a convergent mixed method design [15, 16]. A retrospective medical review utilises pre-existing data originally collected for purposes other than research, such as medical records, and was deemed appropriate for addressing the research question in this study [20]. Research within health care employs quantitative methods to examine causality, generalizability, and effect size, while qualitative methods explore underlying mechanisms, theory development, and individual experiences. The convergent mixed method design draws upon the strengths of both approaches, offering a robust framework for addressing complex challenges in health services ([4, 15]). In this project, we take a pragmatic stance. We recognise that medical records can be seen as showing measurable aspects of patient care, and clinical notes, reflecting clinicians’ interpretations and constructions. This allows us to integrate different forms of knowledge in order to answer the research question.

### Sample and setting

Data were collected in a medium-sized region in southern Sweden, comprising three psychiatric hospitals and approximately 370,000 inhabitants. The region comprises five general psychiatric inpatient wards, including six designated BA rooms, and approximately 150 BA agreements, of which 122 involve women. BA rooms are specifically allocated for BA users and are furnished to provide a homelike environment, including a lockable medication cabinet for storing personal medications. The wards are staffed by mental health assistants, registered nurses, and physicians; however, during BA admissions, no physician consultations or medication adjustments occur. Although the wards are locked, BA users may leave and return as they wish. In the region, access to BA does not require a specific diagnosis. Instead, eligibility is determined based on the patient’s present needs, in mutual agreement between healthcare professionals and the patient. As part of a larger ongoing project, patients have been recruited to the project since 2018. Upon recruitment, patients were informed that over the years, their psychiatric medical records would be reviewed to extract data on their background demographics, life circumstances, psychiatric condition history, health, and utilisation of BA. At the time of data collection, 70 of the 150 patients 70/150, 47% holding a BA agreement had provided written informed consent to participate in the study. Thus, medical records from 70 patients were included. Four medical records were excluded as the patients died after consenting to participate but before the data collection. In total, 66 medical records were reviewed, all of which were psychiatric records from the time the patient was admitted to adult psychiatric care. During the period covered by the BA contracts, 45 patients alternated between different forms of occupation, including work training, employment, sick leave, and participation in daily activities. Four maintained permanent employment throughout the period, while 17 were not engaged in any occupational activity. For the demographic characteristics of the patients whose medical records were reviewed, see Table 1.

**Table 1:** Background demographic characteristics of the patients whose medical records were reviewed

Variable (N = 66)	
Male	7
Female	59
Age M (range years)	32 (20–64)
*USE of BA*	
Duration of access to BA	mean 3 years (range 7 months-6 years)
Did not use BA at all	49%
Used BA once annually	23%
Used BA 2–3 times annually	15%
Used BA 4–5 times annually	11%
Used BA more than 6 times annually	3%
*USE of other care experiences*	
No previous experiences of inpatient care	14%
Previous experience of inpatient care	86%
Extensive experience of inpatient care	
(more than 10 admissions annually in addition to BA)	8%
More than 3 compulsory admissions	15%
Experiences of violence at some point in life	77%
*Diagnoses (range from 1–8 diagnoses)*	
More than 4 diagnoses	55%
Borderline personality disorder	62%
Recurrent depression	50%
ADHD and/or autism	42%
PTSD	38%
Made at least one suicide attempt	89%
Somatic complaints	50%
Menstrual-related issues	33%
Overweight	24%
Chronic pain	21%
Diabetes	15%
*Received treatment*	
Received some form of psychological therapy	85%
Received DBT	45%
Underwent electroconvulsive therapy	38%

**Fig. 1:**
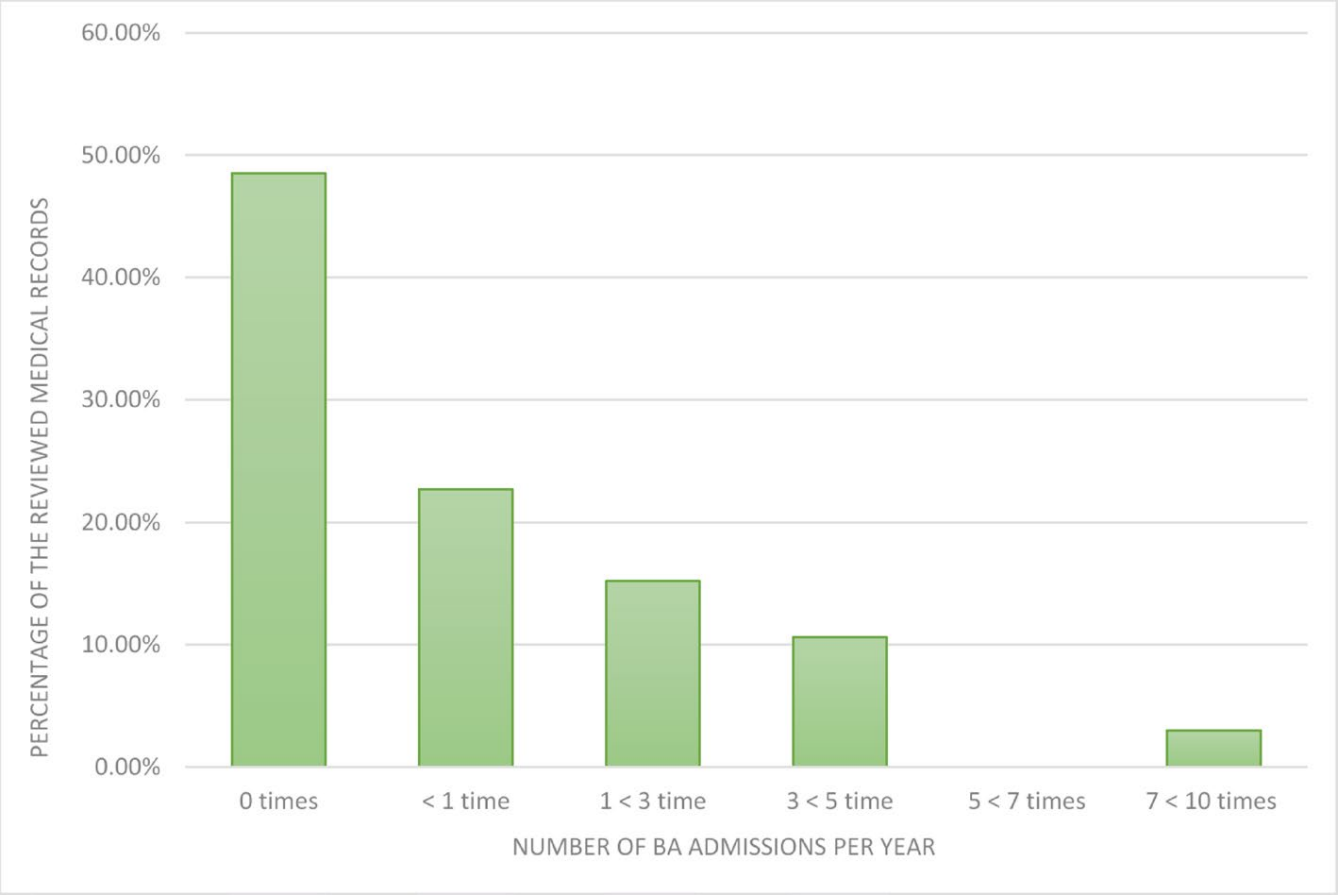
Patients’ use of BA

Among those whose medical records were included in the study, there was considerable variation in the extent of BA use (See Fig. 1).

### Data collection

The medical record review was conducted between June and December 2024. The main aim was to identify information related to utilising BA and potential reasons for its use or non-use. The first author, ET, manually reviewed each patient’s medical record to map the utilisation of BA and to extract information regarding factors influencing the patient’s decision to utilise or refrain from using BA. The medical records were read from the beginning when the patient first entered psychiatric care, through to the most recent entry. For medical records containing up to 300 entries, each note was read in full. For medical records exceeding 300 entries, filters were applied to limit the material (e.g., notes from outpatient visits and entries authored by staff responsible for BA). This limitation was implemented to reduce the volume of material while still aiming to obtain a comprehensive and realistic understanding of the patient, without risking the omission of important content. BA admissions were identified using a procedural code and counted manually. The duration of each patient’s access to BA was determined from the document in which the agreement had been scanned. This document also provided information about the patient’s goals for BA. For patients who no longer had access to BA at the time of review, relevant notes indicating the termination of the agreement or discharge from the clinic were used to calculate the total duration of access.

### Analysis

In a convergent mixed-methods design, qualitative and quantitative data are collected and analysed within the same timeframe, enabling the findings from one method to inform and refine the other in an iterative process [15]. In this study, the qualitative phase of data analysis was implemented first, followed by the quantitative phase. To analyse the qualitative data, a conventional content analysis approach was applied [21]. This inductive method allows categories to be derived directly from the text, rather than based on predefined theoretical frameworks [30].

In the initial phase, background information was extracted from medical records to provide an overview of the patients’ demographic and clinical characteristics. These variables included age, gender, occupational status, experiences of inpatient care, psychiatric diagnoses, treatments, suicide attempts, exposure to violence, and somatic complaints (Table 1). Subsequently, the medical records were systematically reviewed to obtain information regarding the utilisation of BA, including documented reasons for both its implementation and non-implementation. Furthermore, additional contextual factors, such as life circumstances potentially influencing the utilisation of BA, were identified and considered. Clinical notes relevant to the study aim were transcribed verbatim into a separate document to support ongoing interpretation.

Text segments relevant to the study aim were condensed and coded based on their core meaning. Similar content was grouped into preliminary subcategories, enabling differentiation among various phenomena observed in the patient records. These subcategories were developed collaboratively by ET and SH, who jointly agreed on their names and definitions. The categories were anonymised and entered into a Microsoft Excel spreadsheet to facilitate further analysis.

After approximately half of the medical records had been reviewed, recurring patterns and statements began to emerge. The remaining medical records were then reviewed with these subcategories in mind, and earlier records were revisited to ensure consistency and completeness across the dataset. When new information emerged that did not align with existing subcategories, new subcategories were created. During this process, it became apparent that patients’ attitudes towards BA significantly influenced their engagement with the intervention. To explore this further, a keyword search for “brief admission” was conducted across all medical records.

In the final stage of the analysis, ET, SH and RW compared the subcategories and their content, merging or refining them into broader, overarching categories that captured recurring themes. This process resulted in the identification of two final categories. These categories were summarised using frequency and percentage data, based on how many of the 66 medical records reflected each category [15]. The analytical process was dynamic and iterative, involving both a manual review of clinical records and keyword searches. This approach ensured thorough validation of the findings within each category and helped minimise the risk of overlooking relevant data [21].

## Declarations

### Etics

The Swedish Ethical Review Authority in Linköping approved the study [No: 2022–01530–02]. The study and all methods used was conducted in accordance with the guidelines and regulations outlined in the World Medical Association Declaration of Helsinki—Ethical Principles for Medical Research Involving Human Subjects (World Medical Association Declaration of Helsinki, [66]). To protect patients’ personal data, the General Data Protection Regulation (GDPR) was followed [61].

### Concent to participate

Written and oral informed consent was obtained from all patients. They recieved both verbal and written information about the aim of the study, the voluntary nature of their participation, and their right to withdraw at any time without providing a reason, before written informed consent was obtained. The information provided stated that the study would be published in a peer-reviewed international scientific journal. No patients under the age of 16 were included in the study.

### Consent to publish

Not Applicable. We adhered to the *Consolidated criteria for reporting qualitative research (COREQ)* in both the conduct of our study and the presentation of the data [60]. Clinical trial number: not applicable. Data available only on request due to privacy/ethical restrictions.

## Results

The results, derived from the patients’ medical records, are presented in two categories. ‘Circumstances that increase the likelihood of utilising BA’ and ‘Circumstances that decrease the likelihood of utilising BA’, with associated subcategories (see Table 2). It should be noted that the results do not reflect the patient’s own voice, but rather the healthcare professionals’ documentation in which the patient’s thoughts and experiences are conveyed.

**Table 2:** Categories and subcategories of utilisation of BA, based on the retrospective medical review

Category	Subcategory
Circumstances that increase the likelihood of utilising BA	Building trust in BA Applying effective emotional regulation strategies Engaging in meaningful leisure activities Promoting mental health through self-care routines
Circumstances that decrease the likelihood of utilising BA	Experiencing negative encounters with BA Struggling with deservingness and identifying appropriate moments for BA Facing challenges with emotional regulation Managing financial constraints Difficulties Maintaining daily routines

### Circumstances that increase the likelihood of utilising BA

The medical records revealed a range of everyday strategies that patients found supportive in managing their mental health through the use of BA. These included: Building Trust In BA, Applying Effective Emotional Regulation Strategies, Engaging In Meaningful Leisure Activities and Promoting Mental Health Through Self-Care Routines.

### Building trust in BA

A positive attitude towards BA was found in 47% of the patients’ medical records, evenly distributed between those who had used the intervention and those who had not. In some cases, this favourable view was directly associated with BA itself, while in others it reflected more general sentiments. Most patients with a positive attitude described BA as helpful and emphasised the support received from ward staff. One patient valued the autonomy and personal involvement in care that BA facilitated. Others cited a general trust in the healthcare system, and some described the ward as a safe space, irrespective of whether the admission was via BA or a conventional inpatient stay. The majority of patients in the sample, 83%, had the goal of using BA to prevent self-harm.

A total of 26% of patients aimed to use BA to prevent deterioration and to obtain a change of environment or a break from everyday life. Additionally, 8% sought to avoid long-term hospitalisation, while an equal proportion aimed to interrupt other forms of self-destructive behaviour besides self-harm. Among the 49% who had not utilized BA, 63% perceived BA as a source of security in itself. At the same time, some patients referred to the benefits of inpatient care more generally, based solely on their experiences with standard admissions. In several medical records, a sense of control gained from having access to BA was evident.

### Applying effective emotional regulation strategies

According to the medical records, 40% of patients reported having helpful and effective emotional regulation strategies. Of these, 59% stated that they used skills learned through DBT, such as the “opposite action” technique or mindfulness. Established crisis plans were described as helpful in 33% of the medical records, with most plans including BA as one of the steps. In addition, in 15% of the medical records, other anxiety management strategies were described. The ability to implement such strategies increases the likelihood of utilising BA. Conversely, the need for BA may diminish if patients can employ other emotion regulation techniques, such as recognising early warning signs of deterioration and, drawing on lived experience, knowing how to act accordingly, with BA potentially serving as a safety net even when it is not actively utilised.

### Engaging in meaningful leisure activities

Among the patients, 67% were engaged in some form of occupational activity, including work training and daily activities. Approximately 25% described having helpful leisure activities or being able to engage in meaningful ways that supported their mental well-being. Physical activity or exercise was specifically mentioned in 16% of the medical records as a means of promoting health. Physical and leisure activities mentioned in the medical records included horse riding, painting, playing games, and watching television. Additionally, in 9% of the medical records, the social support and sense of community that patients gained through their faith were described as health-promoting. The medical records revealed the benefits of being able to engage in leisure activities while on the ward. It was further highlighted that the BA structure allowed patients to bring personal belongings, enabling them to continue their hobbies during admission, which may have increased the utilisation of the benefits of leisure activities.

### Promoting mental health through self-care routines

The medical records revealed that patients sought BA to receive support with their self-care needs. This was a recurring pattern among those who had previously utilised the intervention. Self-care may encompass support with establishing and maintaining daily routines in various situations and communicating one’s needs to others, both within healthcare settings and in private life. One-fifth (21%) of the medical records illustrated that routines, such as those related to sleep, meals, and physical activity, were helpful in managing self-care and well-being. Some patients described the loss of these routines as life-altering, while others noted that maintaining routines made it easier to cope with mental health challenges. In the medical records, patients stated that they received support with their self-care routines, and their sleep improved while they were admitted to BA.

## Circumstances that decrease the likelihood of utilising BA

The medical records highlighted a range of challenges that could hinder patients from utilising BA. These included: Experiencing Negative Encounters with BA, Struggling with deservingness and identifying appropriate moments for BA, Facing Challenges with Emotional Regulation, Managing Financial Constraints, and Difficulty Maintaining Daily Routines.

### Experiencing negative encounters with BA

A total of 77% of the medical records illustrated negative attitudes either directly towards BA or indirectly in ways that involved BA. The distribution of these attitudes was evenly split between those who had utilised BA and those who had not. Approximately 38% of patients reported a reluctance to be admitted due to previous negative care experiences. Some perceived BA negatively, having associated the ward with past suicide attempts. Others expressed fear that a BA admission might lead to involuntary hospitalisation. Several patients described negative interactions with other inpatients as a reason for avoiding BA, and some noted that their condition worsened during BA due to the influence of others’ poor mental health. In several medical records, patients expressed anxiety about being refused BA admission when seeking help. In a number of the medical records, it was stated that the patients lacked trust in the healthcare system or in psychiatric services. In approximately 18% of patients’ medical records, it was reported that the attitude and behaviour of healthcare staff created barriers to seeking BA. Some felt they were not taken seriously, while others described feeling invisible or ignored by staff, and noted a lack of staff availability during their stay. One patient reported not feeling welcome on the BA ward. Additionally, some described how the attitude of other healthcare professionals (outside the BA unit) also discouraged them from seeking BA. Some medical records noted that all forms of inpatient care were perceived by patients as prison-like. In several cases, inpatient care was characterised as isolating, confining, and lonely, suggesting that perceived limitations in autonomy and emotional support may have contributed to negative evaluations of the intervention. Approximately 17% of patients indicated that BA was not perceived as beneficial or expressed ambivalence regarding its efficacy.

### Struggling with deservingness and identifying appropriate moments for BA

Approximately 11% of the medical records reported that the main challenge in using BA was identifying the “right moment” to seek it. According to the medical records, the personal responsibility that BA is based on was described as both unfamiliar and challenging for some patients. The sudden and rapid onset of anxiety can make it difficult to manage BA independently, and the requirement to initiate contact, particularly when limited to phone calls, can be overwhelming. Several reported struggling to seek BA, describing it as challenging to take the initiative or to call the ward themselves. In approximately 18% of the medical records, it was found that patients had difficulties asking for help. Some reported struggling with communication, particularly in expressing emotions, as well as experiencing intense self-hatred and an inability to attend to their own needs. Many felt like a burden or believed they did not deserve care, expressing concerns about “taking someone else’s place,” which served as a barrier to seeking BA. Several medical records indicated that high personal standards, perfectionistic tendencies, or difficulties with setting boundaries contributed to various problems. It further appeared in the medical journal that some patients stated that they either felt too well to justify utilising BA or too unwell to manage it. Another explained that when their condition deteriorated, it did so rapidly, requiring emergency care instead. One patient noted that self-harming impulses often emerged late at night, when it was too late to utilise BA. Other reasons for difficulties identifying the right moment for BA included uncertainty about how to seek the intervention, the purpose of the intervention, or unawareness of whether the BA agreement was still valid, since it had not been used for a long time. A sense of uncertainty also emerged regarding whether one remained welcome following incidents during previous BA encounters. Several patients identified family members or staff as their primary sources of social support, often individuals who helped them recognize the appropriate timing for BA or employ other effective strategies. Among the patients, 35% reported having fewer than two social contacts, and loneliness was documented in 29% of medical records. This sense of loneliness stemmed not only from a lack of available support, but also from feelings of isolation even when accompanied by others, which may have reduced the likelihood of receiving support in identifying appropriate moments for and utilizing BA.

### Facing challenges with emotional regulation

In addition to self-harm, 54% of the medical records highlight unhelpful strategies to manage emotions. Among these, 56% reported using alcohol or drugs to cope with emotional distress, while 28% engaged in other forms of self-destructive or high-risk behaviours, and one patient was noted to have significant difficulties with empathy. In some records, clinicians described patients frequently entering dissociative states, while others noted aggression as a means of emotional regulation. As these patients did not manage to follow their crisis plans, BA was often disregarded as a coping option. Impulsivity was exhibited in 16% of the medical records, hindering the ability to apply for BA during periods of deteriorating mental health. Instead, impulsive tendencies frequently led to behaviours like self-harm, which subsequently became a barrier to seeking BA. The medical records revealed that difficulties with emotional regulation may lead to conflicts in close relationships, which were described in 36% of the cases. These issues were documented as contributing to deteriorating mental health and increased frequency of crises.

### Managing financial constraints

Approximately 15% of the patients reported various circumstances that prevented them from seeking BA due to work-related reasons, such as fear of losing their job. Financial difficulties were reported by 28% of the patients, who described negative effects on their well-being, elevated stress levels, and barriers to accessing BA. One patient explicitly stated that she had refrained from seeking BA due to being unable to afford public transport to the facility. Others indicated that financial constraints prevented them from taking time off work, thereby limiting their access to BA.

### Difficulty maintaining daily routines

The most frequently reported aspect, identified in 55% of the medical records, was stress-related difficulties that significantly worsened the patients’ mental state and made it more challenging to maintain daily functioning and employ helpful strategies, including the utilisation of BA. A pronounced lack of energy was reported in 10% of the medical records, making it difficult for patients to care for themselves, maintain routines, or seek care, factors that may reduce the likelihood of utilising BA. In the medical records, various contributing factors that could hinder the ability to utilise BA were reported. The medical record review revealed that 43% of patients reported that sleep difficulties negatively affected their daily lives, making it difficult to perform and engage in daily activities. Daily routines, such as caring for family members or pets, could also influence the ability or motivation to utilise BA. There was a struggle to find balance in the daily routines, which in turn increased their stress levels. Notably, in all medical records, BA was utilised by patients as a strategy to understand their own health and care needs and maintain daily routines.

## Discussion

This study highlights several interrelated phenomena that appear to influence the patients’ decisions to utilise or refrain from BA. These include trust in BA, emotional regulation as a mechanism for adaptive care-seeking, engagement in meaningful activities, and support in maintaining self-care routines. Patients were often described in the medical records as viewing BA as a way to prevent self-harm, regain control, and access care in a safe and structured setting. The records also showed that among those who had not utilized BA, 63% perceived BA as a source of security in itself, and that the mere availability of BA could have a preventive function. Conversely, factors that decreased the utilisation of BA use included previous negative experiences with inpatient care, uncertainty about when and how to seek BA, difficulties with emotional regulation, financial constraints, and challenges in maintaining daily routines. Together, these findings illustrate that BA utilisation is not merely a question of access but rather reflects a dynamic interplay between individual capacities, relational trust, and structural conditions. Notably, the data were drawn from medical records and reflect healthcare professionals’ documentation rather than patients’ own accounts, which is important when interpreting how the intervention was understood and used.

An intriguing finding in our study is that nearly half of the patients had never utilised BA, despite having access to it. The sparse use of BA has also been documented in a previous study, where 57% of participants used it only once during a four-year study period [10]. Although the sample size was relatively small in our study (n = 66), this observation merits further reflection. It is not necessarily advantageous for all eligible patients to utilise the intervention, and the fact that approximately 50% did not, may suggest that outpatient care or other life circumstances provided sufficient support, thereby rendering BA unnecessary. As BA is considered a complementary rather than a stand-alone treatment, this may indicate that DBT or other therapeutic approaches have been effective in many cases [, 34, 52, 53].

Moreover, it is well established that patients who engage with BA often present with conditions that exhibit episodic stability. For instance, patients diagnosed with BPD may experience extended periods of psychological well-being [5]. Another plausible explanation for the limited utilisation of BA is that the mere availability of the intervention may offer a sense of reassurance, reducing the perceived need to initiate an admission. The notion that BA agreements can foster a sense of security has been previously documented in the literature [7, 9, 63].

Another possible explanation for why half of the patients never utilised the intervention may be related to challenges in managing their self-care needs. Previous research has documented that many patients receiving psychiatric care face significant challenges in managing their self-care [42]. Translating intention into action may therefore pose challenges for individuals who recognise a need for support, such as BA. Self-care constitutes a dynamic and inherently subjective process that evolves throughout the lifespan and across the trajectory of illness [54]. Variations in self-care behaviours emerge over time, influenced by situational contexts, disease progression, and age-related transitions.

However, modest utilisation of BA may reflect that its mere availability offers psychological reassurance, reducing the perceived need for activation [7, 9, 63]. BA has been shown to support both self-care and access to broader psychiatric services, especially for those with greater functional impairments [7]. Despite greater symptom severity, this subgroup reported high satisfaction, highlighting BA’s role in fostering autonomy and security. Therefore, BA appears beneficial across varying levels of need, supporting both complex cases 7 and individuals with more stable life circumstances [14]. Structured routines during BA, including meals, hygiene, and physical activity, were perceived as stabilising, with effects persisting post-discharge [13]. BA is also associated with enhanced autonomy and role functioning in everyday life [63] and may strengthen individuals’ confidence in initiating health-promoting behaviours [72].

Among the patients, 67% were engaged in and benefited from some form of occupational activity, including daily activities and work training, and 25% highlighted that leisure activities supported their health. Thus, the role of meaningful leisure and occupational activities emerged as an important aspect of maintaining mental health. In a longitudinal follow-up study, Daukantaité et al [7]. demonstrated the importance of having a meaningful everyday life, obtaining employment, and living in a comfortable home environment as factors that can support the individual’s recovery process and reduce the need for BA. In a narrative review, Fancourt et al. [14] identified 600 mechanisms of action through which leisure engagement can affect health and health behaviours. These mechanisms can be categorised as psychological, biological, social, and behavioural processes that operate at individual, group, and societal levels. Engagement in leisure activities can therefore lead to perceived benefits of action and positive outcomes, which in turn increase the likelihood of re-engagement in such behaviours and, over time, reduce engagement in unhealthy behaviours [14, 51, 72 ]. In this study, it was evident that the BA structure allowed patients to bring personal belongings, enabling them to continue their hobbies during admission and thus remain engaged in leisure activities and enjoy their associated health benefits, thereby maintaining a sense of normalcy and well-being. During BA, patients are granted access to personal belongings, including items such as shoelaces and headphones, which are typically restricted in other psychiatric settings [37]. In addition, they retain the autonomy to enter and leave the ward on their own initiative throughout the admission period. This flexibility facilitates the continuation of everyday routines, enabling many patients to attend school or engage in work-related activities while admitted [13, 37, 38]. Each time an individual successfully navigates a situation and experiences a positive emotional response, their ability to handle similar situations is strengthened, increasing the likelihood of repeating the behaviour. This is a key mechanism for changing unhealthy behaviours into healthier ones [51]. Being allowed to maintain leisure activities and independence while on BA can promote health-promoting behaviours [22, 51, 72] and contribute to normalisation within a psychiatric clinical context. Participation and meaning-making are also central components of person-centred and recovery-oriented care [65].

The findings of this study indicate that many patients have limited social networks, with 35% reporting fewer than two supportive contacts. Furthermore, several patients highlighted family members or staff as their primary sources of social support, often those who assist them in seeking BA. Social relationships, including encouragement from friends and family members, positively influence help-seeking by fostering a sense of care and facilitating timely utilisation of BA [22]. The autonomy and voluntary nature of BA can strengthen patients’ sense of agency, allowing them to actively participate in their care and recovery [9, 19]. However, this study also highlights a dynamic tension between patient autonomy and reliance on support from others to utilise BA. Social networks, such as families and close friends, share a life world with the patient that is unique yet interdependent, and mental health professionals must attend to the lived experiences of both patients and their families [6]. Previous research has shown that families often experience anxiety and maintain constant vigilance regarding the well-being of their loved ones [29]. Access to BA, along with social relationships, which are frequently exposed to significant stress and fear, provide hope, support the maintenance of daily routines and are perceived as vital for improving relationships and daily functioning [23, 24, 31, 41]. At the same time, prior negative experiences with BA may undermine trust and motivation to utilise BA [1, 7, 12, 13, 23, 24, 41, 63]. Social networks may share the patient’s concerns, particularly if they have witnessed involuntary inpatient care or prior hospitalisations [28]. It is therefore important to be aware that BA may risk reactivating trauma or negative care associations, even though it is intended to promote a sense of control and safety. Given this history, BA can be challenging and emotionally demanding for both patient and social relations. However, it may also facilitate recovery and healthier relational functioning if professionals provide sensitive support to help social relations adapt to the patient’s self-admission [31]. BA may provide hope for the future while supporting the maintenance of daily routines for both patients and relatives [23, 24, 39]. It is essential to ensure that patients are met with respectful and supportive attitudes from staff, and that BA beds are available,otherwise, patients and relatives may feel neglected or betrayed, discouraging timely help-seeking [22, 23, 24, 31, 39]. Effective self-management requires fundamental skills in self-awareness, decision-making, and problem-solving at a level appropriate to the patient’s abilities and needs [51, 72]. For patients with limited social support, it is important to recognize that loneliness is associated with an increased risk of cognitive decline and premature mortality (WHO, [68]). Loneliness also adversely affects mental health, doubling the likelihood of depression and contributing to anxiety, as well as increasing the risk of self-harm or suicidal ideation. In this context, outpatient staff may play a critical role in facilitating the utilization of BA among individuals with limited social networks. Such support may, in turn, promote positive changes in relational dynamics [1, 31], a pattern that was also evident in the present medical record review.

In our study, 28% of patients reported financial difficulties, describing adverse effects on their well-being and barriers to accessing BA. This underscores the structural vulnerabilities that shape the everyday lives of individuals with psychiatric conditions. Economic constraints, such as the inability to take sick leave or afford transportation, directly limited access to BA. Facilitating transportation has been shown to increase care engagement and reduce missed appointments, particularly among psychiatric patients [62], suggesting that offering similar transport support could improve BA utilization. In Sweden, non‑emergency medical transport is available for somatic healthcare when patients cannot travel by ordinary means due to health reasons, indicating a potential model for psychiatric services. People living with psychiatric disorders are disproportionately affected by financial hardship due to higher rates of poverty, unemployment, underemployment, and dependence on public assistance programmes with strict income and asset thresholds [25, 55]. It emerged from multiple medical records that many experienced difficulties with both their financial situation and employment, which posed significant challenges to seeking and utilising BA. Thus, it is important for healthcare professionals to recognize that the relationship between mental health and employment is multifaceted and influenced by social support, access to care, coping strategies, and economic circumstances [2]. The burden on patients becomes disproportionately heavy when living with an illness for which they cannot receive adequate care due to limited financial resources, a situation consistent with WHO’s observation that social determinants such as employment, education, and housing profoundly shape health outcomes and life expectancy (WHO, [69]).

## Limitations

There are several limitations associated with using a retrospective medical chart review and a convergent mixed method design. As with all retrospective research, the quality of the findings is dependent on the availability and accuracy of the recorded data. The analysis also carries a risk of subjectivity, particularly in the identification of recurring themes. Medical records capture only a fraction of the information shared during clinical encounters, and what is documented depends on the discretion of the healthcare professional. Additionally, documentation practices vary between individuals, resulting in inconsistencies in the level of detail and relevance across medical records.

Not all records could be reviewed in full, which introduces the possibility of data loss during collection [16]. In some cases, patients had over 2,000 entries, making it unfeasible to examine every note. To minimise the risk of missing relevant data, the medical records were searched multiple times using the keyword “brief admission” to capture entries specifically related to the intervention. One limitation of searching medical records this keyword is that the corresponding code in the journal system was introduced after the implementation of BA, potentially leading to incomplete data capture. To minimise the risk of missing patients’ BA stays prior to the introduction of the code, an extensive manual search of medical records was carried out. Findings from the medical journals closely aligned with the calculations regarding the frequency of each theme, thereby demonstrating effective triangulation [15]. For example, the calculations revealed that nearly 50% of patients had utilised BA, while the medical records offered various explanations for refraining from BA. These findings are further supported by existing research on BA [10, 13, 63].

From a theoretical standpoint, positive experiences associated with engaging in health-promoting behaviours are thought to increase the likelihood of repeated engagement [51]. This notion is supported by the medical records, which suggest that BA fosters a sense of control, supports the maintenance of daily structure, and facilitates the continuation of leisure activities that contribute to enhanced well-being [1, 7, 12, 13, 9, 18, 22, 37, 40].

The study is constrained by its small sample size, the absence of a control group, and reliance on clinicians’ journal entries. These limitations were acknowledged and partially mitigated through methodological triangulation; however, they still restrict the generalizability of the findings [15]. By providing a detailed account of the study’s contextual conditions, the findings may offer a degree of transferability to similar settings [50]. Although medical records have inherent limitations as a data source [20], they provide clear information on the number of admissions, the stated goals and utilisation of BA, and general insights into patients’ psychiatric challenges and life circumstances.

Furthermore, the patients with access to BA typically have extensive and ongoing contact with mental health services, which increases the likelihood of obtaining a comprehensive picture through the medical records due to the large volume of available information [3]. The challenges identified in this study are echoed in other recent publications [22, 63], suggesting a shift towards a more balanced understanding of BA. In any case, the results underscore the importance of continued critical examination of both the intervention and the methods used to evaluate it.

### Conclusion

Nearly half of all patients have never utilised BA, though some report that mere access to BA enhances their well-being. Maintaining meaningful activities during the BA stay supports positive, purpose-driven behaviours both in the care setting and at home. Challenges in engaging with BA may increase awareness of mental health and foster self-regulation, particularly when the experience is positive, promoting behavioural persistence and the development of adaptive habits.

Despite BA’s aim to provide a safe and health-promoting environment, certain experiences, such as resurfacing negative experiences from patients or relatives, can hinder its use. Patients with BA agreements may face complex, interrelated challenges, including financial strain, limited social support, and challenges in maintaining daily routines. This highlights the need for comprehensive and individualised strategies to enable the effective utilisation of BA to the extent required. Given that many patients have few social contacts and social support is an important way of assisting patients seeking BA, future efforts should focus on involving individuals already present in the patient’s environment. Outpatient staff can play a key role in supporting patient well-being and identifying when BA or other interventions may be appropriate.

## Data Availability

Data available only on request due to privacy/ethical restrictions.
